# Accuracy of rapid radiographic film calibration for intensity‐modulated radiation therapy verification

**DOI:** 10.1120/jacmp.v7i2.2202

**Published:** 2006-05-25

**Authors:** Ravi Kulasekere, Jean M. Moran, Benedick A. Fraass, Peter L. Roberson

**Affiliations:** ^1^ Department of Radiation Oncology University of Michigan Medical Center Ann Arbor Michigan 48109‐0010 U.S.A.

**Keywords:** radiographic film, IMRT, quality assurance

## Abstract

A single calibration film method was evaluated for use with intensity‐modulated radiation therapy film quality assurance measurements. The single‐film method has the potential advantages of exposure simplicity, less media consumption, and improved processor quality control. Potential disadvantages include cross contamination of film exposure, implementation effort to document delivered dose, and added complication of film response analysis. Film response differences were measured between standard and single‐film calibration methods. Additional measurements were performed to help trace causes for the observed discrepancies. Kodak X‐OmatV (XV) film was found to have greater response variability than extended dose range (EDR) film. We found it advisable for XV film to relate the film response calibration for the single‐film method to a user‐defined optimal calibration geometry. Using a single calibration film exposed at the time of experiment, the total uncertainty of film response was estimated to be <2% (1%) for XV (EDR) film at 50 (100) cGy and higher, respectively.

PACS numbers: 87.53.‐j, 87.53.Dq

## I. INTRODUCTION

With the increased use of radiographic film dosimetry for intensity‐modulated radiation therapy (IMRT) treatment verification,^(^ ^1^ ^–^ ^4^ ^)^ a quick and reliable method of generating film response calibration is desirable. Due to the various contributing errors in film dosimetry (e.g., film manufacture variability, processing conditions variability, densitometer readout errors, and film energy dependence), optimal accuracy of film response requires accompanying calibration films. Traditionally, film sensitometric curves have been performed using standard setup geometries (e.g., $10\times 10\;{\text{cm}}^{2}$ fields exposed at the depth of beam calibration) on a single film for each dose level. The emphasis was placed on accuracy of dose delivery rather than accuracy of film response interpretation. Such calibration techniques are at best inefficient, consuming as many as 15 films to generate a film sensitometric curve, and at worst inappropriate for the exposure geometry. Errors due to film‐to‐film variation and differing scatter conditions can affect the calibration curve when using single fields per film.

Recently, Childress et al.^(1)^ reported on a method of using a single calibration film for IMRT quality assurance (QA) measurements. This method of generating a calibration film saves time and radiographic resources. The use of a single film eliminates film‐to‐film variation errors in the calibration curve and reduces the scatter response by using a $3\times 3\;{\text{cm}}^{2}$ field rather than the $10\times 10\;{\text{cm}}^{2}$ field. Film response differences are minimized by matching scatter conditions, that is, field size and depth in phantom. The use of $3\times 3\;{\text{cm}}^{2}$ fields is also appropriate for IMRT single port verification since IMRT fields are usually delivered as a sum of small field segments. Potential disadvantages of using a single film are (1) response error resulting from the $3\times 3\;{\text{cm}}^{2}$ field being off‐axis instead of being along the central axis of the beam; (2) the cross scatter and transmission effects of irradiating many fields on a single film; and (3) IMRT fields, even though built up from smaller IM fields, can have a potentially significant scatter component due to the summation from all neighboring fields.

In several studies, thin lead shields have been used to attenuate scattered radiation to minimizing film response differences attributed to excess low‐energy scattered photons (scatter filtering).^(^ ^4^ ^,^ ^5^ ^)^ Scatter filtering does attenuate the low‐energy photons but also attenuates a fraction of the high‐energy photons and creates an additional unwanted scatter component. An alternative approach is to minimize error by matching scatter conditions for the calibration to the experiment as closely as possible while keeping the film development and analysis process consistent. Scatter condition matching is particularly useful when the experiment is confined to a single plane at a known depth, as in IMRT QA measurements.

Here, we report on verification measurements performed for the implementation of a variant of the single‐film method suggested by Childress et al.^(1)^ and without the introduction of scatter filtering. We also investigate and discuss film response differences for the single‐film eight‐field method as compared to the standard film calibration method using $10\times 10\;{\text{cm}}^{2}$ fields on multiple films.

## II. METHODS AND MATERIALS

**Figure 1:**
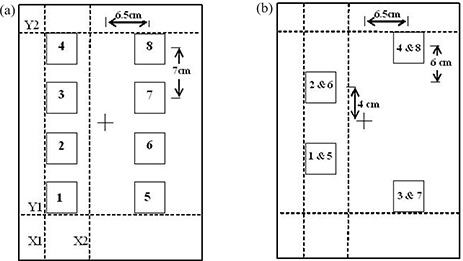
Irradiation pattern and jaw collimator positions (Y1, Y2, X1, X2) at the start of the irradiation process for the left‐hand field pattern. After the first segment, the jaw is closed over position 1 for the second segment, etc. The field pattern on the right of the film was performed in a separate step by resetting the jaw and MLC positions. Shown are (a) an eight‐field single‐film pattern and (b) a four‐field two‐film pattern.

Eight $3\times 3\;{\text{cm}}^{2}$ fields of escalating dose were delivered to a single film (Fig. 1(a)) and to two films (Fig. 1(b)), each with four $3\times 3\;{\text{cm}}^{2}$ fields placed strategically to minimize scatter, on Kodak X‐OmatV (XV) and extended dose range (EDR) film placed at a 5‐cm depth in Solid Water (Gammex RMI Model 457). Backscsatter was provided with 10 cm of Solid Water material to match the clinical IMRT QA geometry. The two‐film method reduced cross‐field scatter at the expense of time and materials working with two rather than one film.

The eight‐field pattern (Fig. 1(a)) was exposed at 5 cm depth in the phantom at isocentric distance, as a single treatment, using the X and Y jaws and a step‐and‐shoot auto‐multileaf collimator (MLC) technique from a 6‐MV Varian Clinac 2100 EX beam. The fields and collimator positions were designed to minimize collimator transmission and cross‐field scatter. Each field was a $3\times 3\;{\text{cm}}^{2}$ square with the centers displaced 7 cm along the radial axis and 6.5 cm on each side along the transverse axis. During the initial irradiation sequence, the Y‐jaws were placed symmetrically at the 24 cm opening, and the X‐jaws were placed such that the inner jaw was set at the minimum allowed −2cm (across the midline) and the outer jaw setting was at +8cm. After the initial irradiation of four squares, the MLC leaves as well as the (lower) Y1‐jaw closed over the first square (Y1jaw=+5cm) for the irradiation of the remaining three squares. This process was repeated including the Y1 jaw movement, giving the four squares ascending dose levels. The right side was irradiated similarly, except the initial irradiation of all four squares received a greater dose. The irradiation sequence was very similar to that followed by Childress et al.^(1)^ except that here the jaws as well as MLC were used to block out the squares that had already received the planned dose. The four‐field, two‐ film exposures were performed similarly (Fig. 1(b)).

The dynamic range covered by each type of calibration film was chosen to encompass the dose range typically used with clinical IMRT treatment fields. The dose levels selected were 16 cGy to 128 cGy in increments of 16 cGy for the XV film and double that dose for the EDR film. The dose ranges encompassed the most likely useful ranges for each film for IMRT validation measurements.

The actual dose delivered to each of the fields, including scatter and transmission, was measured using an Exradin Model‐14 microchamber (a collecting volume of 0.009 cm^3^ and a cross‐sectional area of 0.09 cm^2^) and an Innovision Model‐35040 electrometer with automatic leakage correction. The ion chamber was placed in a predrilled cavity at the midpoint of a 2‐cm thick Solid Water slab. Dose measurements were performed with an additional 9‐cm Solid Water for backscatter and 4‐cm Solid Water for buildup and attenuation. Accurate placement of the ion chamber in the cavity was verified with radiographic film. Care was taken to avoid errors due to stem leakage by placing as much of the chamber stem as possible outside the field of view of direct beam. Stem leakage measurements were performed to verify that corrections to the measured dose values were not required. Ion chamber response was calibrated at the isocenter using the standard calibration geometry of $10\times 10\;{\text{cm}}^{2}$ field at 90 cm source‐to‐surface distance (SSD) and 10‐cm Solid Water buildup and backscatter. The ion chamber measurements were performed for representative geometries for each position for all irradiation conditions. Measurements for some positions under a jaw were estimated with sufficient precision from measurements for symmetric geometries. Automatic leakage correction was turned off, and manual leakage corrections were performed for measurements performed under a jaw.

Film measurements were taken using XV and EDR type film. The film was placed in Solid Water at 95 cm SSD (5‐cm buildup) with 10‐cm backscatter. The film irradiations used a perpendicular configuration. A pinhole was punched on the corner of the film pack to release trapped air. The scatter and transmission contributions were incorporated into the calculation of the monitor units (MUs) necessary to deliver the expected dose levels for each field. The eight‐field calibration film response was compared to the film response from $10\times 10\;{\text{cm}}^{2}$ field exposures at depths of 10 cm and 5 cm. The effects of field size (output factor) and depth were verified using ion chamber measurements for the $3\times 3\;{\text{cm}}^{2}$ and $10\times 10\;{\text{cm}}^{2}$ fields. Measurements were performed to investigate the film response to scatter conditions due to the off‐axis placement of the $3\times 3\;{\text{cm}}^{2}$ fields in the eight‐field calibration film as well as the effect of the processing and digitizing variations. XV film measurements for a $3\times 3\;{\text{cm}}^{2}$ field constructed with MLC only, MLC/jaws, and off‐axis MLC/jaws placed as in the eight‐field calibration film were performed and compared to the response for the $3\times 3\;{\text{cm}}^{2}$ central axis jaw field. The MUs delivered to each position were adjusted to give the same dose (~128cGy) to the center of the field.

All exposed films were processed within a few hours following irradiation to minimize processing errors using a Kodak X‐Omat 3000RA film processor. In order to maintain the processor within a given specification, a quality control (QC) program was set up using a multivariate statistical process control (MSPC) method. This process involved exposing a standard sensitometric strip onto a film and then comparing the film response of three preselected levels on the strip to established mean values. Optical density was measured using a Nuclear Associates Model 07–024 Digital Densitometer. The statistical values for the MSPC program were established using data collected over several weeks to include film batch variation and daily machine output variations. Film processing was performed only if the measured optical density (OD) values for the three locations on the sensitometric strip were within 2 standard deviations from the mean. If not, flashed and preprocessed films were fed through the processor to allow the developer chemistry to reach proper equilibrium conditions through replenishment until the processor passed the QC criteria. Processed films were digitized using a Lumisys Lumiscan 75 (also known as Kodak LS75) laser digitizer, which was calibrated for horizontal uniformity using the film step wedge supplied by the manufacturer. Interdate and intradate variations in the eight‐field films were measured to allow estimation of the total error in the film process.

Gray level values for each exposed film were extracted using a 0.18 cm^2^ area at the center of the exposed $3\times 3\;{\text{cm}}^{2}$ field using the University of Michigan treatment planning software (UMPLAN). A standardized Kodak film step wedge was used to convert the gray levels to OD. A single unexposed film from the same film batch was also processed and digitized with the calibration film to use as the base/fog value for each experiment.

## III. RESULTS

The irradiation process outlined in the Methods section for the eight‐field calibration film was performed in less than 10 min and was found to be useful in minimizing dose from scatter and MLC leakage.

The ion chamber measurements used to calculate the number of MUs required to deliver the appropriate dose to each square are given in Table 1. The ion chamber was placed at the center of each field position (see Fig. 1 for position sequence). The full eight‐field calibration sequence was used for either 200 MUs for in‐field readings or 600 MUs for transmission‐ and scatter‐only readings. Some measurements for transmission‐ and scatter‐only positions were not taken by appealing to symmetry and realizing that 10% precision was sufficient (i.e., positions with one or possibly two significant digits in Table 1). The dose values (and MUs) obtained for each position on the calibration film were doubled for use with EDR film.

**Table 1:** Ion chamber measured data (cGy/MU) at each field position with all eight fields irradiated used to calculate the monitor units (MU) required to generate the predetermined dose levels at each position on the XV film calibration exposure. All values were doubled for EDR film exposure.

Field		1	2	3	4	5	6	7	8	Dose (cGy)
detector position	1	0.8890	0.0015	0.0012	0.0002	0.0023	0.0012	0.0005	0.0001	16.3
	2	0.8877	0.8749	0.0035	0.0007	0.0029	0.0020	0.0011	0.0004	32.1
	3	0.8917	0.8881	0.8752	0.0025	0.0030	0.0029	0.0015	0.0006	48.2
	4	0.8943	0.8900	0.8873	0.8734	0.0027	0.0021	0.0013	0.0008	64.1
	5	0.0023	0.0012	0.0005	0.0001	0.8906	0.0015	0.0012	0.0002	80.3
	6	0.0029	0.0020	0.0011	0.0004	0.8946	0.8803	0.0035	0.0007	95.7
	7	0.0030	0.0029	0.0015	0.0006	0.8973	0.8937	0.8803	0.0025	112.0
	8	0.0027	0.0021	0.0013	0.0008	0.8992	0.8962	0.8904	0.8762	128.1
MUs		18	18	18	18	90	17	18	18	

**Figure 2:**
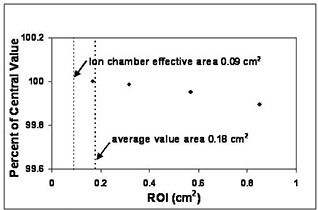
Average optical density (OD) for the region of interest (ROI) as a percent of the central value for position 6 of the eight‐field calibration film (XV).

Optical density readings for a square of area 0.18 cm^2^ (compared to the 0.09 cm^2^ cross‐sectional area for the sensitive volume of the Exradin Model 14 microchamber) were averaged to provide a reasonable sample (~1150pixels) to reduce random error and minimize effects due to minor film imperfections. The average response for the $3\times 3\;{\text{cm}}^{2}$ field decreases as the area of the region of interest (ROI) is increased. The average response as a percent of the field center and as a function of the measured ROI for position 6 of the XV calibration film is shown in Fig. 2. A 1 cm^2^ ROI can be sampled in the $3\times 3\;{\text{cm}}^{2}$ field without introducing a systematic error greater than approximately 0.2%.

**Figure 3:**
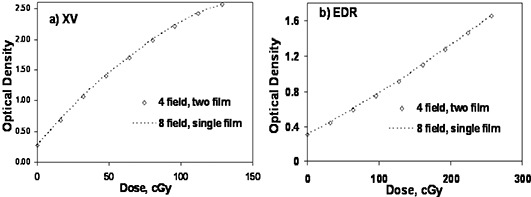
Comparison of single‐film eight‐field calibration and the two‐film four‐field calibration for (a) XV film and (b) EDR film. The dashed line represents the eight‐field technique. Film was exposed at 5 cm depth and 95 cm SSD.

A comparison between the eight‐field single film and four‐field dual film calibration method is shown in Fig. 3. The dose levels (and MUs) for the four‐field method were measured using a microchamber using a technique similar to the eight‐field case discussed above. At the 50 cGy (100 cGy) level for the XV (EDR) film the difference between the single‐film and two‐film methods is less than 2% (1%). The data show that there is no significant advantage in favor of using the four‐field, two‐film method over the eight‐field single‐film method. The four‐field method will not be discussed further.

**Figure 4:**
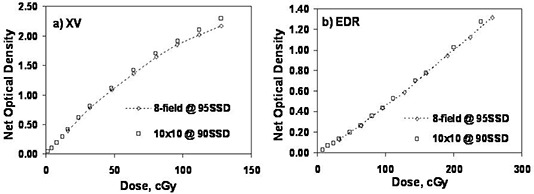
Comparison of results for the eight‐field method exposed at 5 cm depth with the standard calibration conditions of $10\times 10\;{\text{cm}}^{2}$ field at 10 cm depth for (a) XV film and (b) EDR film. The dotted curve is a polynomial fit through the eight‐field data.

A standard film calibration method (optimized for minimum uncertainty in delivered dose) uses a $10\times 10\;{\text{cm}}^{2}$ field at the calibration depth of 10 cm. Figure 4 shows comparisons of film response to the standard calibration method with the eight‐field calibration film at a depth of 5 cm. The dotted line is a second‐order polynomial fit to the eight‐field calibration film response data. For both XV and EDR film the agreement in the low‐dose region is very good. However, the film response to the $10\times 10\;{\text{cm}}^{2}$, depth of 10 cm, field was greater at higher doses compared to the $3\times 3\;{\text{cm}}^{2}$ eight‐field response. The response was 4% (2%) greater at a dose level of 50 cGy (100 cGy) for XV (EDR) film.

**Figure 5:**
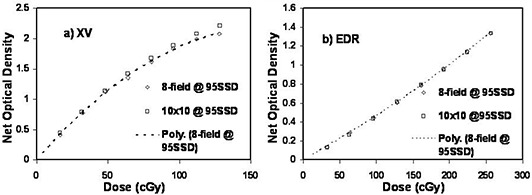
Comparison of the eight‐field method with standard single $10\times 10\;{\text{cm}}^{2}$ field per film method exposed at 5 cm depth for (a) XV film and (b) EDR film.

Figure 5 shows the comparison between the $10\times 10\;{\text{cm}}^{2}$ calibration method and the $3\times 3\;{\text{cm}}^{2}$ eight‐field calibration method both exposed at 95 cm SSD and 5 cm depth for XV and EDR film. The XV film again shows greater response to the $10\times 10\;{\text{cm}}^{2}$ field in the higher dose regions. The EDR response was not significantly different. The $10\times 10\;{\text{cm}}^{2}\;\text{XV}\;(\text{EDR})$ calibration film response was 2% (0.5%) greater for a dose of 50 cGy (100 cGy) compared to the eight‐field calibration film response at the same depth of 5 cm.

To investigate the depth and field size effects on film response, an experiment was conducted using XV film at depths of 5 cm and 10 cm for $3\times 3\;{\text{cm}}^{2}$ and $10\times 10\;{\text{cm}}^{2}$ jaw fields on the central axis. Prior to exposing the film, the output factors for each field size and depth were obtained using an Exradin A‐14 microchamber in Solid Water and verified by comparison with the accelerator commissioning data. The number of MUs needed was adjusted for each field size and depth to deliver approximately the same dose at the center of the field. Films were exposed to three levels of dose covering the range of doses given to the calibration films. The data obtained for the $10\times 10\;{\text{cm}}^{2}$ field at 10 cm depth was fitted to a polynomial. The slope of the polynomial was used to correct for small discrepancies in the delivered dose caused by monitor unit round‐off. There was a 2% response difference between the $3\times 3\;{\text{cm}}^{2}$ field at 5 cm depth and the $10\times 10\;{\text{cm}}^{2}$ field at 10 cm depth for XV film at dose levels above 50 cGy. This difference did not fully explain the ~4% difference observed between the eight‐field calibration film and the $10\times 10\;{\text{cm}}^{2}$, 10 cm depth, film response at 50 cGy described above for XV film.

To further investigate the response difference, XV‐film response for central axis exposures of a $3\times 3\;{\text{cm}}^{2}$ field created using the jaws (with dose levels verified by ion chamber measurements) were compared to the results of the eight‐field method. The eight‐field method film response was 1% (50 cGy) and 0.7% (100 cGy) less than the $3\times 3\;{\text{cm}}^{2}$ field at the central axis. The measurements performed to investigate the film response to the off‐axis placement of the $3\times 3\;{\text{cm}}^{2}$ fields in the eight‐field calibration film traced the bulk of the discrepancy to the position on the film, not the position of the off‐axis irradiation or the transmission‐scatter exposure conditions. The data showed that the response variation due to the (quality‐controlled) processor across the film surface was up to 1%. The digitizer response was somewhat variable, but XV flood film measurements demonstrated that the maximum systematic deviation of ~1% for XV film was present at the off‐axis $3\times 3\;{\text{cm}}^{2}$ positions used for the eight‐field calibration technique compared to the central film position used for central axis exposures. The cumulative effect was a 1% to 2% variation for XV film dependent on processor conditions (~random) and calibration of the film digitizer (~systematic). This level of position‐dependent random and systematic error is also present in the routine IMRT QA verification measurements using XV film.

**Figure 6:**
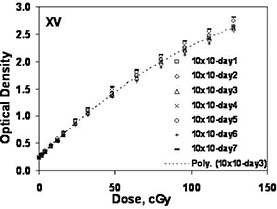
Interdate variation for calibration films generated using XV film and single $10\times 10\;{\text{cm}}^{2}$ central axis fields exposed at 90 SSD and 10 cm depth.

Figure 6 shows the interdate variation of calibration films (XV) taken with the $10\times 10\;{\text{cm}}^{2}$ single field per film (15 film) method. Data shown were obtained over a period of several months, and the data for day 3 were fitted to a simple polynomial. The standard deviation in the film response at 50 cGy was 4.6% for the seven measurements.

Table 2 shows the interdate variation of eight‐field XV and EDR calibration films based on a two‐month time period (six or seven films, respectively). EDR film response variations were similar at the low dose levels, but were approximately half as much at higher dose levels. Intradate variability was sampled for XV using nine films processed consecutively. The standard deviations of the film responses ranged from 1% at the lowest dose levels to 0.7% at the highest dose levels. Intradate variability for EDR film was observed to be less than for XV film and was not considered to be a significant source of measurement error (~0.5%).

**Table 2:** Interdate film error estimates over a two‐month period

XV Film		EDR Film	
Dose (cGv)	Percent standard deviation	Dose (cGv)	Percent standard deviation
16	7.2%	32	7.6%
32	5.3%	64	3.3%
48	5.1%	96	2.9%
64	4.6%	128	2.4%
80	4.0%	160	2.1%
96	4.0%	192	1.8%
112	3.9%	224	1.9%
128	3.7%	256	1.4%

In summary, the response differences observed between the eight‐field single‐film (5 cm depth) method and the $10\times 10\;{\text{cm}}^{2}$ multifilm (10 cm depth) method was due to exposure conditions (2% to 3% for XV film) and processor/digitizer error (1% to 2% for XV film). To achieve the desired calibration conditions at minimum measurement uncertainty using XV film dosimetry, it is necessary to use a measured response correction for the eight‐field, single‐film method. Measurement error observed for EDR film was significantly less, making the use of a response correction less compelling.

## IV. DISCUSSION

Rapid radiographic calibration using a single‐film, eight‐field method is advantageous for clinical IMRT dose verification, compared to calibration films generated by single $10\times 10\;{\text{cm}}^{2}$ fields on multiple films. The single‐film process realizes a total time savings of about 2 h in generating a film sensitometric curve for IMRT QA over the conventional multifilm method. Using an eight‐field pattern with jaws and MLC reduces the scatter and transmission component over an MLC‐driven technique. We have shown the film errors to be manageable using a jaws plus MLC method while avoiding the need of introducing thin lead shields for an IMRT QA application. Introducing thin lead shield has the disadvantage of attenuating the primary beam as well as generating additional scatter when the high‐energy photons interact with the high *Z*material in the lead filter. In addition, the method of exposing the eight‐field calibration film as a single pattern using jaws plus MLC was time‐efficient, taking less than 10 min to deliver.

Using a two‐film, four‐field per film pattern to generate the eight‐field calibration film did not present obvious advantages over the single‐film eight‐field pattern. The reduction in the scatter‐ and transmission‐induced film response was minimal between the two methods. The following discussion focuses mainly on the single‐film eight‐field calibration technique.

Response variations of radiographic film to the changing geometric exposure conditions are well known.^(^ ^6^ ^,^ ^7^ ^)^ For XV film, we observed ~3% increased response in the sensitometric curve between the standard $10\times 10\;{\text{cm}}^{2}$ field exposure and the $3\times 3\;{\text{cm}}^{2}$ eight‐field exposure (with processor response correction) at a dose level of 50 cGy. For EDR film the increased response was ~2%. These response differences were traced to the effects of depth and field size. The increased response was likely due to in‐phantom scatter altering the photon spectra responsible for the film exposure.^(7)^ The EDR film showed lower response differences, attributed to the reduced density of silver halide atoms compared to XV film. The processor and digitizer can adversely affect the response, particularly (as in this case) when the response calibration and experiment are performed using different physical positions on the film.

If a calibration film is exposed for each film IMRT film measurement, the remaining film response error is dependent on intradate processing error, digitizer error, and spectral matching error. Intradate processing error can be minimized using a film processor QA program. Digitizer error can be due to random readout error and systematic response error. The systematic error may be film‐dependent,^(1)^ but can be controlled by periodic recalibration or correction factor measurement. Spectral matching error can be minimized by measurement matching or spectral filtering.^(4)^

The ideal calibration film response for IMRT QA should account for small‐field effects superimposed on large‐field phantom scatter effects. The eight‐field, single‐film method can mimic a multifield calibration method by measurement of response levels to determine a systematic adjustment to form an effective dose versus response curve. The advantage of a single‐film calibration can be achieved for any defined ideal calibration curve. The ideal curve should match the spectral level of the IMRT field, which probably varies between the conditions for a $3\times 3\;{\text{cm}}^{2}$ to $10\times 10\;{\text{cm}}^{2}$ field at the QA exposure depth. The changing spectral composition over complex IMRT fields, or from one IMRT field to another, limits the ability of the choice of calibration geometry to perfectly reflect the correct spectra. Thus, EDR film response being less dependent on the geometric exposure conditions would be less error‐prone.

It is essential that tight control is maintained on the film processor. In this instance the processor was required to pass an established QA process before any dosimetric film was processed. The error introduced by the processor can be easily corrected since it is systematic. This leads to the possibility of having a dynamic film calibration process. In such a process only certain points along the curve that show the greatest susceptibility to the processor variation are measured and corrected based on an established curve. This will save additional time in the calibration process since the full curve does not have to be measured each time. Processor variation‐induced errors amounted to ~1% or less of the total error in film dosimetry in our experiments (less for EDR than XV). With appropriate internal corrections applied to the horizontal nonuniformity, the error introduced by the digitizer can also be maintained to <1%.

## V. CONCLUSIONS

Single‐film exposure film dosimetry to support IMRT QA can be performed with reasonable error (~2% or less) without the use of lateral scatter filtering. Estimated film errors indicate that EDR film has less overall error than XV film. However, for practical situations XV film response is acceptable for dose levels up to 100 cGy and is preferable if dose levels below 50 cGy are of interest. EDR film is recommended for higher dose levels. Implementation of film processor QA procedures using statistical process control methods reduces the systematic error in film dosimetry.

## References

[1] Childress NL , Dong L , Rosen II . Rapid radiographic film calibration for IMRT verification using automated MLC fields. Med Phys. 2002;29:2384–2390. 12408313 10.1118/1.1509441

[2] Dogan N , Leybovich LB , Sethi A . Comparative evaluation of Kodak ERD2 and XV2 films for verification of intensity modulated radiation therapy. Phys Med Biol. 2002;47:4121–4130. 12476986 10.1088/0031-9155/47/22/314

[3] Chetty IJ , Charland PM . Investigation of Kodak extended dose range (EDR) film for megavoltage photon beam dosimetry. Phys Med Biol. 2002;47:3629–3641. 12433124 10.1088/0031-9155/47/20/305

[4] Ju SG , Ahn YC , Huh SJ , Yeo IJ . Film dosimetry for intensity modulated radiation therapy: Dosimetric evaluation. Med Phys. 2002;29:351–355. 11929018 10.1118/1.1449493

[5] Burch SE , Kearfott KJ , Trueblood JH , Sheils WC , Yeo JI , Wang CK . A new approach to film dosimetry for high energy photon beams: Lateral scatter filtering. Med Phys. 1997;24:775–783. 9167171 10.1118/1.597999

[6] Danciu C , Proimos BS , Rosenwald JC , Mijnheer BJ . Variation of sensitometric curves of radiographic films in high energy photon beams. Med Phys. 2001;28:966–974. 11439493 10.1118/1.1376443

[7] Palm A , Kirov AS , LoSasso T . Predicting energy response of radiographic film in a 6 MV x‐ray beam using Monte Carlo calculated fluence spectra and absorbed dose. Med Phys. 2004;31:3168–3178. 15651599 10.1118/1.1812911

